# Cervical aortic arch in the pediatric population: a meta-analysis of individual patient's data

**DOI:** 10.3389/fcvm.2023.1266956

**Published:** 2023-09-28

**Authors:** Massimo Baudo, Alessandro Varrica, Matteo Reali, Antonio Saracino, Mario Carminati, Alessandro Frigiola, Alessandro Giamberti, Mauro Lo Rito

**Affiliations:** ^1^ Department of Congenital Cardiac Surgery, IRCCS Policlinico San Donato, San Donato Milanese, Italy; ^2^ Department of Cardiac Surgery, ASST Spedali Civili di Brescia, University of Brescia, Brescia, Italy; ^3^ Department of Pediatric and Adult Congenital Cardiology, IRCCS Policlinico San Donato, San Donato Milanese, Italy

**Keywords:** cervical aortic arch, congenital heart disease, cardiac surgery, pediatrics, meta-analysis

## Abstract

**Background:**

This is the first meta-analysis to analyze all reports of published pediatric cases of cervical aortic arch (CAA) by highlighting the clinical characteristics and treatment outcomes using the reported individual data of the patients. The aim of the study is to investigate the clinical features and surgical outcomes of such a rare disease in the pediatric population.

**Methods:**

A comprehensive search was conducted in various academic databases, including PubMed, ScienceDirect, SciELO, DOAJ, and Cochrane Library, until June 2022 for case reports describing the presence of cervical aortic arch in the pediatric age. Case reports and series were included if the following criteria were met: (1) description of the cervical aortic arch; (2) patient of pediatric age; and (3) published in the English language. All other types of publications that lacked patient-specific information were excluded from the analysis. This systematic review was conducted in accordance with the PRISMA guidelines. The primary outcome measure of the analysis was early and late mortality.

**Results:**

The literature search identified 2,272 potentially eligible articles, 72 of which met our inclusion criteria with 96 patients including the author's institutional case. At a median of 365 (90–730) days, the overall cohort registered a 7.3% (7/96) mortality rate. In the subset of patients who underwent surgery, the mortality rate was also 7.3% (4/55), and the mortality rate following surgery to treat only CAA was 2.4% (1/42). Dyspnea was identified as an independent determinant of mortality by employing the univariable Firth bias-reduced logistic regression method.

**Conclusion:**

Cervical aortic arch is a rare congenital heart disease that poses treatment challenges due to the high anatomical variability, diverse clinical presentations, and the presence of other concomitant diseases. The surgical treatment appears to be a safe and effective approach for resolving the symptoms, although it needs to be tailored individually for each patient.

**Systematic Review Registration:**

https://www.crd.york.ac.uk/prospero/display_record.php?RecordID=346826, Identifier: CRD42022346826.

## Introduction

The cervical aortic arch (CAA) is a relatively rare congenital anomaly of the aorta development in which the aortic arch is located above the superior aspect of the clavicle, occasionally protruding high into the neck. CAA was initially introduced by Reid in 1914 (1), and the number of recorded cases in literature remains relatively low. CAA may be associated with other structural anomalies (such as kinking, coarctation, or aneurysm) or congenital heart disease (CHD), or it can occur as an isolated cardiovascular anomaly (2). Aortic arch anomalies exhibit a higher prevalence among patients with chromosome 22q11 deletion, whether associated with heart malformation or not (3).

Embryologically, the arch normally arises from the fourth branchial arch. It is theorized that in cases of CAA, the arch arises in a more cephalad location from the second or third branchial arch, resulting in an elevated final position of the arch (4).

Most often asymptomatic, CAA can manifest as a swelling pulsatile mass at the base of the neck where a murmur can be heard or a thrill can be felt. In cases when it is observed, signs and symptoms are associated with the presence of a vascular ring that compresses the trachea or esophagus (i.e., stridor, dyspnea, recurrent pulmonary infections, or dysphagia) (5).

**Figure 1:**
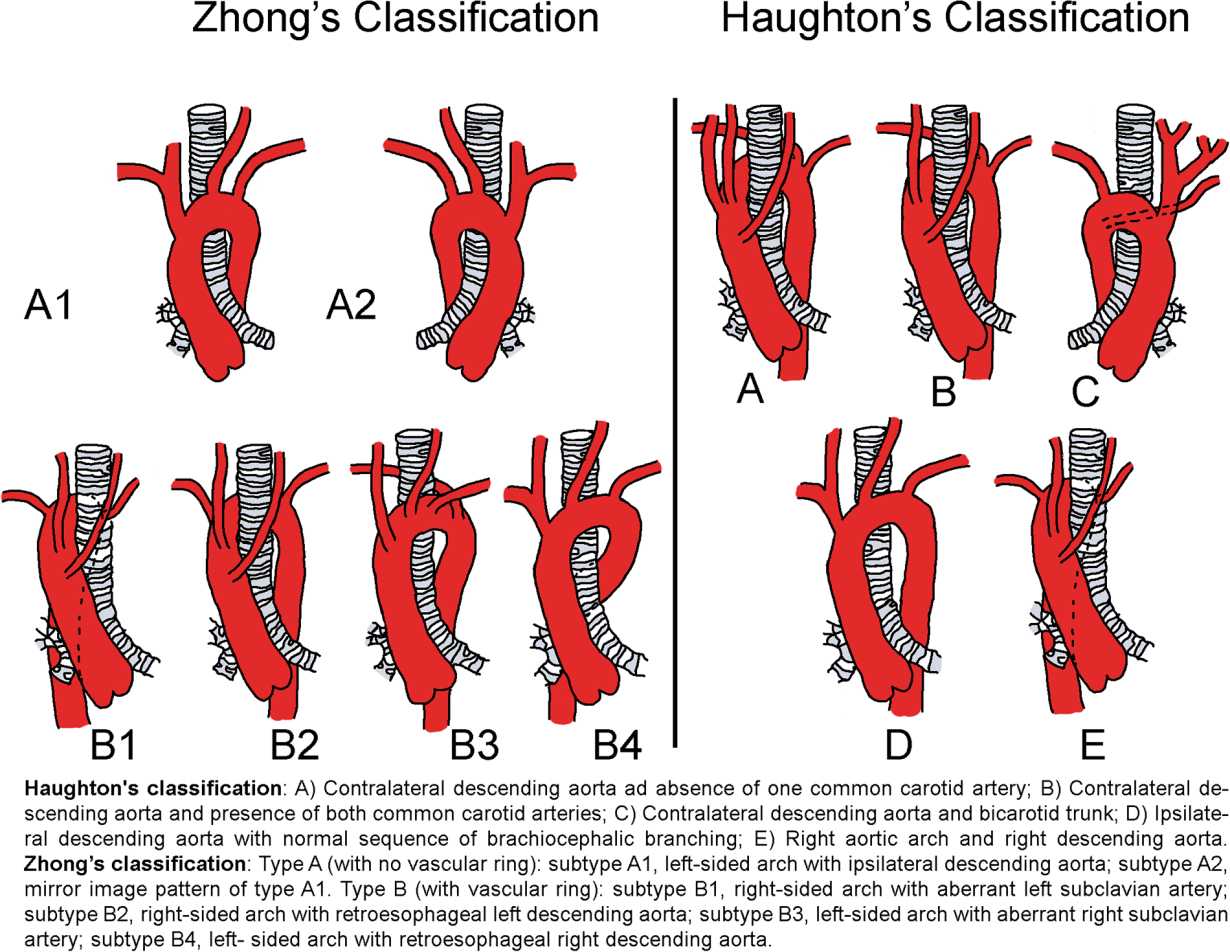
Haughton and Zhong cervical aortic arch classification diagram.

Historically, Haughton et al. (6) proposed the first classification of CAA in 1975, encompassing five morphological types (A–E) based on their own observations and a review of the available literature cases. More recently, Zhong et al. (7) proposed a revised classification of CAA in an attempt to provide a more intuitive classification that could be used for surgical decision making. The classification consists of two types and six subtypes on the basis of the presence of a vascular ring (i.e., retroesophageal aortic segment and/or aberrant subclavian artery) and the relationship of the descending thoracic aorta to the side of the aortic arch (Figure 1).

The existing reports on CAA are currently constrained to studies involving small cohorts and case reports. In the present study, we seek to provide the most comprehensive review of pediatric cases and evaluate the demographics, clinical presentation, and surgical procedures of patients of pediatric age with CAA.

## Materials and methods

### Literature search strategy

**Figure 2:**
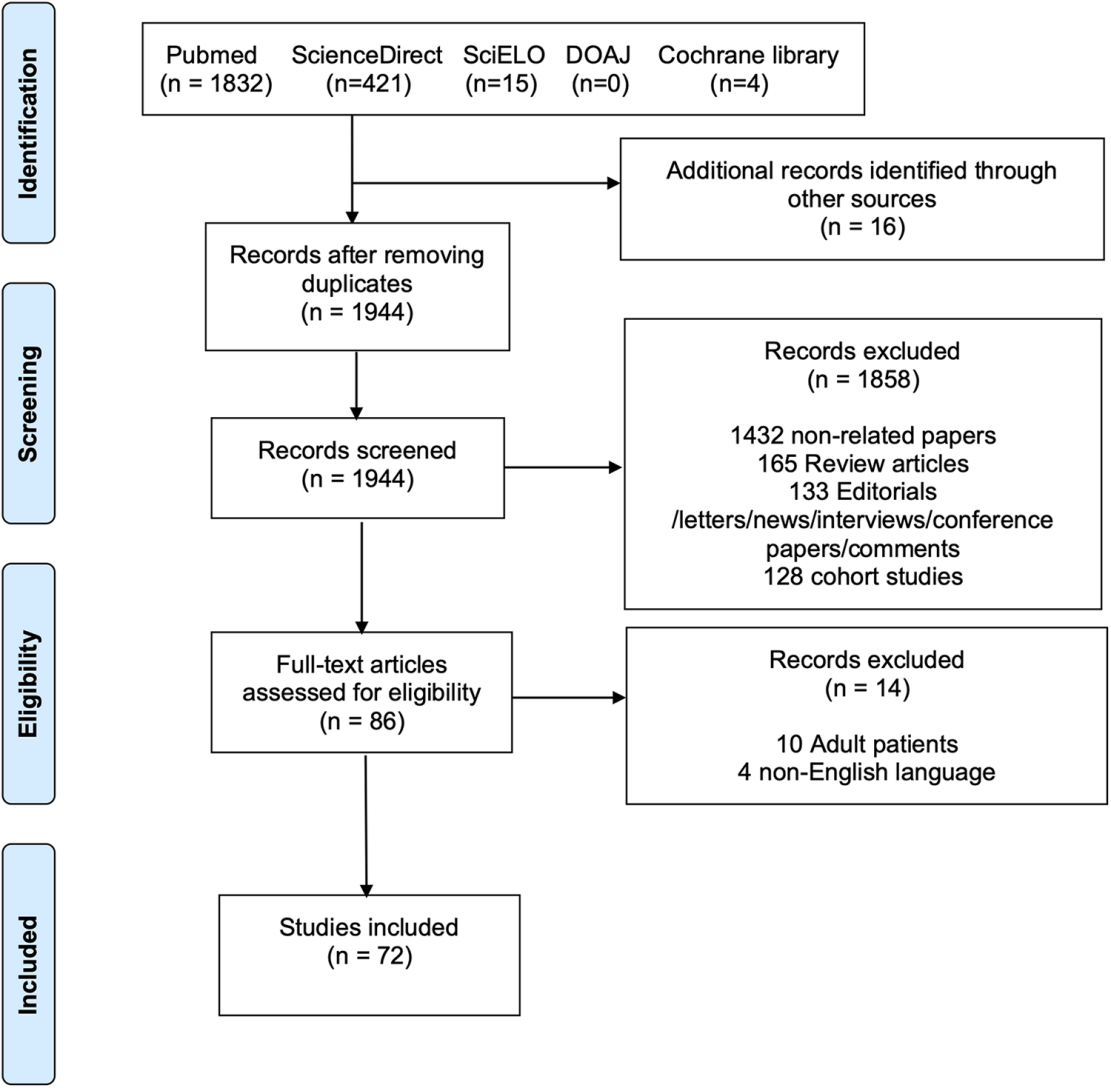
PRISMA flow diagram of the included studies. The following steps were taken for study selection: (1) identification of titles of records through database search; (2) removal of duplicates; (3) screening and selection of abstracts; (4) assessment for eligibility through full-text articles; and (5) final inclusion in the study.

This systematic review was conducted in accordance with the PRISMA (Preferred Reporting Items for Systematic Reviews and Meta-Analyses) guidelines (see the Supplementary Material) (8). The PRISMA flow diagram is presented in Figure 2. PubMed, ScienceDirect, SciELO, DOAJ, and Cochrane Library databases were searched until June 2022 for case reports and series describing the presence of cervical aortic arch in the pediatric age. The complete search strategy is shown in Supplementary Table S1. Furthermore, the references of all studies and meta-analyses were examined to identify additional articles (i.e., “backward snowballing”). The study selection process involved the following steps: (1) identification of titles of records through database search; (2) removal of duplicates; (3) screening and selection of abstracts; (4) assessment for eligibility through full-text articles; and (5) final inclusion in the study. Two authors (MB and MLR) independently screened the studies for inclusion. Discrepancies were arbitrated by a third author (AG) to achieve consensus.

This review was registered with the PROSPERO register of systematic reviews (ID: CRD42022346826). For the systematic review, data were obtained from published papers. As such, the approval of the research ethics board or the consent of the patient was not required. Regarding the institutional case report, the consent to use personal data for scientific research purposes was obtained through the signing of the surgical consent form. The data that support the findings of this study are available from the corresponding author upon reasonable request.

### Selection criteria

Using the Population, Interventions, Comparison, Outcome, and Study design (PICOS) strategy, the case reports and series were included if the following criteria were met: (1) description of the cervical aortic arch; (2) patient of pediatric age; (3) published in the English language. Exclusion criteria for analysis were all other forms of publications that lacked patient-specific information.

### Data extraction and critical appraisal

Microsoft Office 365 Excel software (Microsoft, Redmond, WA, USA) was used for data extraction. The following patient characteristics were extracted: age, sex, cervical arch laterality, presence of other aortic defects, concomitant CHD, signs, symptoms, and surgical procedure performed.

The expected differences in the information reported from the cases were observed, and to a certain degree, each article presented distinct variables that were not found in other reports. As a result, the absence of data for certain variables necessitated subjective interpretation, Significant relevant complications that were not reported were assumed not to have occurred. Denominators were determined in the data analysis based on either explicit indications of the presence or absence of a variable, or through suitable inferences of their existence.

The Joanna Briggs Institute Critical Appraisal tool was used for the critical appraisal of the quality of the included case reports (9).

### Statistical analysis

The primary outcome measure of the analysis was early and late mortality, while the secondary outcome measure was to analyze the clinical presentation of CAA. Categorical variables were presented as frequency counts and percentages and compared between groups using the Chi-square test or Fisher's exact test, as required. After assessing the normality of continuous variables using Kolmogorov–Smirnov test, data were presented as means and standard deviations if normally distributed and were compared between groups using Student's *t*-test or analysis of variance (ANOVA). The data were presented as medians and interquartile ranges (IQR) if not normally distributed and compared between groups using Mann–Whitney *U* test or Kruskal–Wallis test, accordingly. Symptom and mortality predictors were identified using univariable Firth bias-reduced logistic regression. The independent predictors of late mortality were evaluated using univariable Cox regression with Firth's correction method. The Firth bias-reduced correction method has become a standard approach for analyzing binary outcomes with small samples and reduces the bias in maximum likelihood estimates of coefficients. All tests were two-sided, and the alpha level was set at 0.05 for statistical significance.

All analyses were performed using R version 4.2.1 (R Project for Statistical Computing, Vienna, Austria) and RStudio version 2022.07.1 Built 554.

## Results

**Figure 3:**
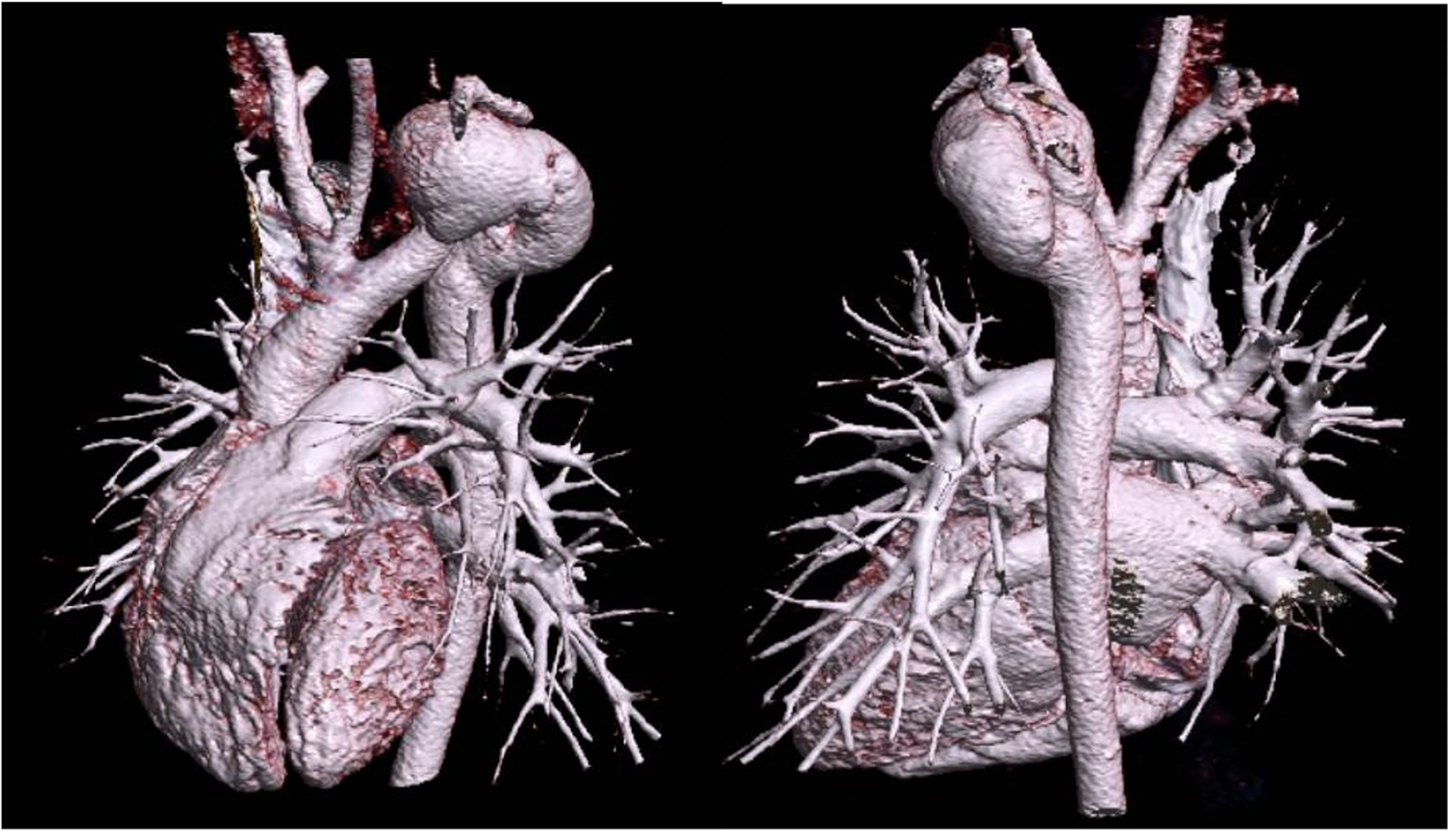
CT scan 3D reconstruction of the author's institutional cervical aortic arch case. 3D reconstructed CT scan showing the left-sided cervical aortic arch anatomy of the institutional patient included in the analysis.

The literature review was conducted adding the authors' institutional case. In brief, a 6-year-old girl was diagnosed with isolated aortic arch aneurysm with surgical indication. The pre-natal cardiologic evaluation revealed the presence of a dilated aortic arch. Marfan syndrome and other collagenopathies were excluded at the genetic evaluation after birth. At the cardiologic follow-up visit, a contrast-enhanced CT scan was performed, revealing the presence of a left CAA aneurysm (max diameter 29 mm) with a branching left subclavian artery (Figure 3). The patient underwent resection of the aneurysm, followed by a termino-lateral anastomosis with an anterior autologous pericardium patch. In addition, the subclavian artery was reimplanted into the ascending aorta using a 6 mm vascular conduit through a median sternotomy. The postoperative hospital stay was uneventful, and the patient was discharged on the sixth postoperative day. At the last follow-up visit, she was found to be alive and in good health.

### Study selection and characteristics

The literature search identified 2,272 potentially eligible articles. Additional 16 articles were identified using the backward snowballing method. After removing the duplicates, 1,944 papers were screened. A total of 86 full-text articles were assessed for eligibility, and 72 publications (6, 10–80) were found to meet our inclusion criteria (Figure 2) with 95 patients. The authors’ institutional case was added to the study population. Thus, a total of 96 patients were finally included. The publication year ranged from 1947 to 2021. The critical assessment of the included articles is shown in Supplementary Table S2.

### Meta-analysis

The median age of the general population was 6 years (IQR: 0.83–11) with a female prevalence of 62.8% (54/86). Two-thirds of the CAA were right-sided (64/96, 66.7%), and 86.5% (83/96) of the descending aorta were left-sided. Concomitant arch-associated anomalies and concomitant CHD were present in 69.8% (67/96) and 41.7% (40/96) of the patients, respectively. The most prevalent Haughton class was type B (41/92, 44.6%), while the most prevalent Zhong class was type B (67/92, 72.8%) with B2 being the most prevalent subclass (48/92, 52.2%, Table 1). In total, 46 out of 88 patients (52.3%) were asymptomatic, while the most prevailing symptom reported was dyspnea (21/88, 23.9%, Table 2). In total, 55 out of 93 (59.1%) patients underwent surgery with or without CAA correction, while 42 out of 93 (45.2%) patients underwent surgery to treat CAA. Thus, 13 out of 93 patients (14.0%) did not undergo CAA surgical correction. Of note, there were 27 patients in total that underwent surgery to treat an isolated CAA (including all diameter variations, i.e., aneurysm, coarctation, arch hypoplasia, and double aortic arch). A sternotomy was chosen in 57.9% (11/19) of the cases, while a thoracotomy was preferred in 42.1% (8/19) of the patients. None of these patients died. At a median of 365 (90–730) days, the overall cohort registered a 7.3% (7/96) mortality rate. Three of these cases were related to surgery for treating CHD without the CAA (14, 27, 39), while only one case focused on surgery for isolated CAA (2). The three non-operated patients experienced different causes of death: one patient died due to acute cerebral hemorrhage (6), another patient died due to sepsis with deranged coagulation, renal failure, and cardiopulmonary failure (50), and the third patient experienced unexpected death 24 h after undergoing cerebral arteriography, with no identifiable cause (17). After surgery, only four patients (among the symptomatic), all with a vascular ring, did not completely resolve their symptoms, which persisted in a milder form than prior to intervention (6, 19, 39, 54). More details can be seen in Table 3.

**Table 1:** Baseline characteristics of the included patients overall and divided by sex.

Characteristic	Overall (*n* = 96)	Male (*n* = 33)	Female (*n* = 56)	*p*-value
Age, median (Q1–Q3), years	6.00 (0.83–11.00)	6.00 (1.04–12.5)	7.00 (0.79–11.00)	0.99
Female sex, No. (%)	54/86 (62.8%)	0/33 (0%)	56/56 (100%)	**<0** **.** **001**
Cervical arch laterality, No. (%)				
Left	31/96 (32.3%)	10/33 (30.3%)	18/56 (32.1%)	0.86
Right	64/96 (66.7%)	23/33 (69.7%)	37/56 (66.1%)	0.99
Both	1/96 (1.0%)	0/33 (0%)	1/56 (1.8%)	0.99
Descending aorta laterality, No. (%)				
Left	83/96 (86.5%)	30/33 (90.9%)	47/56 (83.9%)	0.52
Right	13/96 (13.5%)	3/33 (9.1%)	9/56 (16.1%)	
Vascular ring, No. (%)	69/94 (73.4%)	24/33 (72.7%)	43/56 (76.8%)	0.66
Aortic arch-associated anomalies, No. (%)	67/96 (69.8%)	19/33 (57.6%)	45/56 (80.4%)	**0**.**021**
Kommerell's diverticulum	25/96 (26.0%)	8/33 (24.2%)	16/56 (28.6%)	0.66
Coarctation	21/96 (21.9%)	5/33 (15.2%)	11/56 (19.6%)	0.78
Arch aneurysm	11/96 (11.5%)	2/33 (6.1%)	9/56 (16.1%)	0.20
Hypoplastic	6/96 (6.3%)	4/33 (12.1%)	2/56 (3.6%)	0.19
Double arch	5/96 (5.2%)	0/33 (0%)	5/56 (8.9%)	0.15
Kinking	7/96 (7.3%)	3/33 (9.1%)	4/56 (7.1%)	0.71
Interrupted arch	1/96 (1.0%)	0/33 (0%)	1/56 (1.8%)	0.99
Cardiac-associated anomalies, No. (%)	40/96 (41.7%)	17/33 (51.5%)	18/56 (32.1%)	*0*.*07*
Ventricular septal defect	15/96 (16.6%)	8/33 (24.2%)	6/56 (10.7%)	*0*.*09*
Tetralogy of Fallot	7/96 (7.3%)	2/33 (6.1%)	4/56 (7.1%)	0.99
Atrial septal defect	5/96 (5.2%)	4/33 (12.1%)	1/56 (1.8%)	*0*.*06*
Bicuspid aortic valve	4/96 (4.2%)	3/33 (9.1%)	1/56 (1.8%)	0.14
Tricuspid atresia	2/96 (2.1%)	1/33 (3.0%)	0/56 (0%)	0.37
Truncus arteriosus	3/96 (3.1%)	0/33 (0%)	2/56 (3.6%)	0.52
Double outlet right ventricle	1/96 (1.0%)	0/33 (0%)	0/56 (0%)	0.99
Interrupted arch	1/96 (1.0%)	0/33 (0%)	1/56 (1.8%)	0.99
Zhong classification, No. (%)				
A1	22/92 (23.9%)	7/33 (21.2%)	12/55 (21.8%)	0.95
A2	2/92 (2.2%)	2/33 (6.1%)	0/55 (0%)	0.14
B1	7/92 (7.6%)	2/33 (6.1%)	5/55 (9.1%)	0.71
B2	48/92 (52.2%)	17/33 (51.5%)	30/55 (54.5%)	0.78
B3	8/92 (8.7%)	4/33 (12.2)	4/55 (7.3%)	0.47
B4	4/92 (4.3%)	1/33 (3.0%)	3/55 (5.5%)	0.99
Not classifiable	1/92 (1.1%)	0/33 (0%)	1/55 (1.8%)	0.99

Bold means statistically significant.

**Table 2:** Signs and symptoms of the included patients overall and divided by sex.

Characteristic	Overall (*n* = 96)	Male (*n* = 33)	Female (*n* = 56)	*p*-value
Symptoms, No. (%)				
Asymptomatic	46/88 (52.3%)	14/31 (45.2%)	29/50 (58.0%)	0.26
Dyspnea	21/88 (23.9%)	10/31 (32.3%)	6/50 (12.0%)	**0**.**03**
Recurrent RTI	12/88 (13.6%)	6/31 (19.4%)	4/50 (8.0%)	0.17
Dysphagia	7/88 (8.0%)	3/31 (9.7%)	3/50 (6.0%)	0.67
Cough	3/88 (3.4%)	2/31 (6.5%)	1/50 (2.0%)	0.56
Syncope	2/88 (2.3%)	0/31 (0%)	2/50 (4.0%)	0.52
Hemiparesis	1/88 (1.1%)	0/31 (0%)	1/50 (2.0%)	0.99
Chest pain	1/88 (1.1%)	0/31 (0%)	1/50 (2.0%)	0.99
Headache	1/88 (1.1%)	0/31 (0%)	1/50 (2.0%)	0.99
Diplopia	1/88 (1.1%)	1/31 (3.2%)	0/50 (0%)	0.38
Palpitations	1/88 (1.1%)	1/31 (3.2%)	0/50 (0%)	0.38
Signs, No. (%)				
Murmur	45/87 (51.7%)	19/30 (63.3%)	25/50 (50.0%)	0.25
Pulsating mass	36/87 (41.4%)	13/30 (43.3%)	22/50 (44.0%)	0.96
Limbs pressure difference	21/87 (24.1%)	8/30 (26.7%)	8/50 (16.0%)	0.25
Palpable thrill	20/87 (23.0%)	8/30 (26.7%)	12/50 (24.0%)	0.79
Cyanosis	12/87 (13.8%)	5/30 (16.7%)	4/50 (8.0%)	0.28
Stridor	7/87 (8.0%)	4/30 (13.3%)	2/50 (4.0%)	0.19
Underwent surgery	55/93 (59.1%)	19/30 (63.3%)	34/56 (60.7%)	0.67
Sternotomy	16/55 (29.1%)	5/19 (26.3%)	11/34 (32.4%)	0.76
Thoracotomy	17/55 (30.9%)	7/19 (36.8%)	9/34 (26.5%)	0.43
Neck	1/55 (1.8%)	0/19 (0%)	1/34 (2.9%)	0.99
Unknown	21/55 (38.2%)	7/19 (36.8%)	11/34 (32.5%)	0.74

RTI, respiratory tract infection.

Bold means statistically significant.

**Table 3:** Outcomes of included studies divided by sex.

Characteristic	Overall (*n* = 96)	Male (*n* = 33)	Female (*n* = 56)	*p*-value
Underwent surgery for arch, No. (%)	42/93 (45.2%)	12/33 (36.4%)	28/56 (50.0%)	0.21
Sternotomy	16/42 (38.1%)	5/12 (41.7%)	11/28 (39.3%)	0.99
Thoracotomy	12/42 (28.6%)	3/12 (25.0%)	8/28 (28.6%)	0.99
Unknown	14/42 (33.3%)	4/12 (33.3%)	9/28 (32.1%)	0.99
Overall mortality, No. (%)	7/96 (7.3%)	3/33 (9.1%)	4/56 (7.1%)	0.71
Mortality after surgery, No. (%)	4/55 (7.3%)	3/19 (15.8%)	1/34 (2.9%)	0.13
Mortality after surgery for arch, No. (%)	1/42 (2.4%)	1/12 (8.3%)	0/28 (0%)	0.30
Median follow-up, median (Q1–Q3), days	365 (90–730)	287.5 (187.5–652.5)	365 (42–1,095)	0.85

When analyzing sex differences, it was observed that females exhibited a significantly higher prevalence of concomitant arch-associated anomalies (80.4% vs. 57.6%, *p* = 0.021), while experiencing a lower incidence of dyspnea (12.0% vs. 32.3%, *p* = 0.026) compared with males. Females demonstrated a lower prevalence of concomitant CHD (32.1% vs. 51.5%, *p* = 0.071), specifically a reduced occurrence of atrial septal defects (1.8% vs. 12.1%, *p* = 0.061). More details are shown in Table 1.

The patients undergoing surgery were younger when compared with the non-surgical subgroup [4.5 (0.47–10) years vs. 8.0 (2.56–11.25) years, *p* = 0.036]. Furthermore, the surgical patients were more symptomatic compared with the non-operated patients (60.4% vs. 31.6%, *p* = 0.008), particularly those who suffered more from dyspnea (35.4% vs. 2.6%, *p* < 0.001). The operated patients presented significantly more concomitant arch-associated anomalies (80.0% vs. 57.9%, *p* = 0.021), particularly of arch aneurism (18.2% vs. 2.6%, *p* = 0.025), when compared with the non-surgical subgroup. Of note, the overall mortality rate was similar between the two groups (7.3% vs. 7.9%, *p* = 0.999). All details can be seen in Supplementary Table S3.

The cervical aortic arches classified as Zhong type A exhibited a higher tendency for the presence of a CAA on the left side compared with those classified as Zhong type B (84.0% vs. 13.4%, *p* < 0.001). Interestingly, Zhong type A was more prone to show a concomitant arch aneurysm or kinking of the aorta when compared with Zhong type B (36.0% vs. 3.0%, *p* < 0.001 and 20.0% vs. 3.0%, *p* = 0.015, respectively), but with a lower prevalence of Kommerell's diverticulum (8.0% vs. 34.3%, *p* = 0.016) (Supplementary Table S4).

### Regression analysis

The univariable Firth bias-reduced logistic regression analysis revealed that interrupted arch (*p* = 0.017), tricuspid atresia (*p* = 0.049), and dyspnea (*p* = 0.034) were identified as independent determinants of mortality. A higher mortality rate was noted in individuals with right-sided CAA (*p* = 0.057) (Supplementary Table S5).

Kinking of the arch was negatively associated with symptoms (*p* = 0.007), while surgery (*p* = 0.008) was positively associated with symptoms. A positive association between symptoms and concomitant CHD (*p* = 0.062), double aortic arch (*p* = 0.087), and coarctation (*p* = 0.082) was observed. In addition, a negative association was seen between age (*p* = 0.057) and symptoms (Supplementary Table S6).

The results of the univariable Cox regression analysis with Firth's correction revealed that interrupted arch was found to be an independent risk factor for mortality (*p* = 0.014). A lower mortality rate was observed when surgery was performed (*p* = 0.065) (Supplementary Table S7).

## Discussion

To our knowledge, this is the first meta-analysis to analyze all reports of published pediatric cases of CAA by highlighting the demographics, clinical characteristics, and treatment outcomes using the individual data of the patients. Since all the papers were documented as either case reports or case series, they provided a comprehensive of the most relevant data needed.

CAA is a rare congenital anomaly whose embryological origin remains unsolved. Normally, the aortic arch develops from the fourth of the six aortic arches during embryonic development. There are currently two competing theories to explain the CAA variation (4). The first one hypothesizes that the cervical arch arises from the persistence of the third embryonic arch, while the ipsilateral fourth arch regresses. The other theory assumes an anomalous positioned fourth arch that did not undergo the typical descent process.

Two main CAA classifications have been proposed by Haughton et al. (6) and Zhong et al. (7). The classification proposed by Haughton is considered to be a predominantly historical categorization, as it was developed based on a limited number of cases. Zhong's more recent classification can be viewed as a revised classification that was established with a greater emphasis on potential surgical approaches and anatomical traits, i.e., the presence of a vascular ring. Nevertheless, the population used to construct the sample comprised a cohort of young adult patients. The present review of the pediatric population showed how Zhong's classification identifies additional anatomical characteristics apart from the vascular ring in the pediatric population. Zhong type A is associated to more left-sided aortic arches, while type B is associated to more right-sided variants. Moreover, arch aneurysms and kinking are more related to Zhong type A than type B.

Due to the rarity of CAA and the associated complexity with concomitant CHD and arch abnormalities, the surgical management of CAA is technically challenging and remains unstandardized. The aims of surgery encompass the correction of concomitant CHD when present, along with decompression of the esophagus and trachea in the presence of a vascular ring. Therefore, surgical accesses and procedures are tailored individually according to all of these parameters. The selection of the thoracotomy incision side is determined by the CAA laterality, while a sternotomy is generally preferred for cases of higher complexity. By definition, the presence of a vascular ring in Zhong type B CAA implies a more complicated surgery. In fact, thoracotomies were performed twice as frequently in type A CAA as in type B CAA (38.5% vs. 18.5%, respectively), despite the fact that the difference was not statistically significant. However, based on the limited number of reported deaths, it is not feasible to draw conclusions whether distinctions exist among the various surgical strategies.

The regression analysis conducted in the present review provided insights into the prevailing trends regarding surgical interventions, indicating a preference for performing surgery on patients displaying symptoms as opposed to those who were asymptomatic. Within this context, symptomatology, particularly dyspnea, emerged as a prominent factor influencing the decision to proceed with surgical intervention. Moreover, factors indicative of a more severe pathological condition, such as interrupted aortic arch and tricuspid atresia, were identified as independent determinants of mortality. However, this last point warrants further discussion. In the current study population, only two cases of tricuspid atresia and one case of interrupted aortic arch were observed. The patient in the latter case did not undergo surgery and died a few weeks later. Both patients diagnosed with tricuspid atresia had surgical intervention: one patient survived, while the other patient died 1 year after surgery. On the basis of a comprehensive analysis of these statistics and the observation of extremely wide confidence intervals, it becomes evident that the clinical significance of the two identified risk factors is not firmly established. Rather, their influence appears to be primarily statistical in nature, highlighting the need for cautious interpretation.

### Limitations

While the majority of individual data could be retrieved from the published papers, it is important to note that certain minor information was not always present, and this could be a possible source of bias. In addition, the exclusion of non-English studies may introduce a potential source of selection bias. Finally, the limited duration of the follow-up period restricts the scope of the evaluated outcome data.

### Conclusion

CAA is a rare CHD that poses treatment challenges due to the high anatomical variability, diverse clinical presentations, and the presence of other concomitant CHD. The surgical treatment appears to be a safe and effective approach for resolving the symptoms, although it needs to be tailored individually for each patient. Finally, Zhong's classification offers anatomical associations that prove to be useful in informing surgical strategies.

## Data Availability

The raw data supporting the conclusions of this article will be made available by the authors, without undue reservation.
