# Supplementary material for: Cervical aortic arch in the pediatric population: a meta-analysis of individual patient's data
Source: Front Cardiovasc Med. 2023 Sep 28;10:1266956. doi: 10.3389/fcvm.2023.1266956 (PMC10580808; doi:10.3389/fcvm.2023.1266956)
Supplement: Supplementary file 1 [file Datasheet1.docx]

**Supplementary Table 1 - Search strategy**

| # | Search |
| --- | --- |
| 1 | "cervic"[All Fields] OR "cervicals"[All Fields] OR "cervices"[All Fields] OR "neck"[MeSH Terms] OR "neck"[All Fields] OR "cervical"[All Fields] |
| 2 | "aorta, thoracic"[MeSH Terms] OR ("aorta"[All Fields] AND "thoracic"[All Fields]) OR "thoracic aorta"[All Fields] OR ("aortic"[All Fields] AND "arch"[All Fields]) OR "aortic arch"[All Fields] |
| 3 | 1 AND 2 |

**Supplementary Table 2 – Critical appraisal of the included papers**

| **Case Report** | **Q1** | **Q2** | **Q3** | **Q4** | **Q5** | **Q6** | **Q7** | **Q8** |
| --- | --- | --- | --- | --- | --- | --- | --- | --- |
| Adaletli 2007 | Yes | Yes | Yes | Yes | No | No | Yes | No |
| Ahluwalia 2007 | Yes | Yes | Yes | Yes | Yes | Yes | Yes | No |
| Almeida 2003 | Yes | Yes | Yes | Yes | No | No | Yes | Yes |
| Baker 1987 | Yes | Yes | Yes | Yes | No | No | Yes | No |
| Baravelli 2007 | Yes | Yes | Yes | Yes | No | No | Yes | Yes |
| Beavan 1947 | Yes | Yes | Yes | Yes | Yes | Yes | Yes | No |
| Binsalamah 2018 | Yes | Yes | Yes | Yes | Yes | Yes | Yes | No |
| Bisset 1987 | Yes | Yes | Yes | Yes | No | No | Yes | No |
| Bourdon 1981 | Yes | Yes | Yes | Yes | No | No | Yes | No |
| Caputo 2010 | Yes | Yes | Yes | Yes | No | No | Yes | No |
| Chang 1971 | Yes | Yes | Yes | Yes | Yes | Yes | Yes | No |
| Chen H.Y. 2002 | Yes | Yes | Yes | Yes | Yes | Yes | Yes | No |
| Chen F.L. 2008 | Yes | Yes | Yes | Yes | Yes | Yes | Yes | No |
| Chibane 2013 | Yes | Yes | Yes | Yes | Yes | Yes | Yes | No |
| Cornali 1976 | Yes | Yes | Yes | Yes | No | No | Yes | No |
| Costanzo 2014 | Yes | Yes | Yes | Yes | Yes | Yes | Yes | No |
| DCruz 1983 | Yes | Yes | Yes | Yes | No | No | Yes | No |
| Dey 2018 | Yes | Yes | Yes | Yes | No | No | Yes | No |
| Doorenbos 1991 | Yes | Yes | Yes | Yes | No | No | Yes | No |
| Duke 2001 | Yes | Yes | Yes | Yes | Yes | Yes | Yes | No |
| Fernández-Doblas 2012 | No | No | No | Yes | No | No | No | No |
| Floemer 2000 | Yes | Yes | Yes | Yes | Yes | Yes | Yes | No |
| Gerrah 2012 | No | No | No | Yes | Yes | Yes | Yes | No |
| Guha 2016 | Yes | Yes | Yes | Yes | No | No | Yes | No |
| Haliloglu 2007 | Yes | Yes | Yes | Yes | No | No | Yes | No |
| Harley (Lewis) 1959 (1953) | Yes | Yes | Yes | Yes | Yes | Yes | Yes | No |
| Hastreiter 1966 | Yes | Yes | Yes | Yes | No | No | Yes | Yes |
| Haughton 1975 | Yes | Yes | Yes | Yes | No | No | Yes | Yes |
| Hellenbrand 1978 | Yes | Yes | Yes | Yes | Yes | Yes | Yes | No |
| Higuchi 2003 | Yes | Yes | Yes | Yes | Yes | Yes | Yes | No |
| Huang 2007 | Yes | Yes | Yes | Yes | Yes | Yes | Yes | No |
| Hunter 1973 | Yes | Yes | Yes | Yes | Yes | Yes | Yes | No |
| Hyman 1975 | Yes | Yes | Yes | Yes | No | No | Yes | No |
| Jain 2010 | Yes | Yes | Yes | Yes | Yes | Yes | Yes | No |
| Jhaveri 2018 | Yes | Yes | Yes | Yes | Yes | Yes | Yes | No |
| Karakurt 2011 | Yes | Yes | Yes | Yes | No | No | Yes | No |
| Kasar 2018 | Yes | Yes | Yes | Yes | No | No | Yes | No |
| Kazuma 1997 | Yes | Yes | Yes | Yes | No | No | Yes | No |
| Kumar 1989 | Yes | Yes | Yes | Yes | No | No | Yes | No |
| Kumar 1997 | Yes | Yes | Yes | Yes | Yes | Yes | Yes | No |
| Lipchik 1967 | Yes | Yes | Yes | Yes | Yes | Yes | Yes | No |
| Lu 2010 | Yes | Yes | Yes | Yes | Yes | Yes | Yes | No |
| Mahoney 1964 | Yes | Yes | Yes | Yes | Yes | Yes | Yes | No |
| Makani 2017 | Yes | Yes | Yes | Yes | Yes | Yes | Yes | No |
| Massumi 1963 | Yes | Yes | Yes | Yes | Yes | Yes | Yes | No |
| McCue 1973 | Yes | Yes | Yes | Yes | No | No | Yes | No |
| McElhinney 1999-2000 | No | No | No | Yes | Yes | Yes | Yes | Yes |
| Moncada 1975 | Yes | Yes | Yes | Yes | No | No | Yes | Yes |
| Morgan 1976 | Yes | Yes | Yes | Yes | No | No | Yes | No |
| Mullins 1973 | Yes | Yes | Yes | Yes | No | No | Yes | No |
| Nagashima 2010 | Yes | Yes | Yes | Yes | Yes | Yes | Yes | No |
| Ojiha 2013 | Yes | Yes | Yes | Yes | No | No | Yes | No |
| Öztunç 2004 | Yes | Yes | Yes | Yes | Yes | Yes | Yes | No |
| Patel 2009 | Yes | Yes | Yes | Yes | No | No | Yes | No |
| Pearson 1997 | Yes | Yes | Yes | Yes | Yes | Yes | Yes | Yes |
| Priya 2020 | Yes | Yes | Yes | Yes | No | No | Yes | No |
| Rajbanshi 2016 | Yes | Yes | Yes | Yes | Yes | Yes | Yes | No |
| Ramaswamy 2021 | Yes | Yes | Yes | Yes | Yes | Yes | Yes | No |
| Sang 1987 | Yes | Yes | Yes | Yes | No | No | Yes | No |
| Schleman 1975 | Yes | Yes | Yes | Yes | Yes | Yes | Yes | No |
| Shakerian 2015 | Yes | Yes | Yes | Yes | Yes | Yes | Yes | No |
| Shepherd 1969 | Yes | Yes | Yes | Yes | No | No | Yes | No |
| Shuford 1972 | Yes | Yes | Yes | Yes | No | No | Yes | No |
| Takao 1979 | Yes | Yes | Yes | Yes | Yes | Yes | Yes | No |
| Tiraboschi 1980 | Yes | Yes | Yes | Yes | No | No | Yes | Yes |
| Tjang 2008 | Yes | Yes | Yes | Yes | Yes | Yes | Yes | No |
| Vaillant 1988 | Yes | Yes | Yes | Yes | Yes | Yes | Yes | No |
| van Nooten 1986 | Yes | Yes | Yes | Yes | Yes | Yes | Yes | No |
| van Son 1999 | Yes | Yes | Yes | Yes | Yes | Yes | Yes | No |
| Walker 2002 | Yes | Yes | Yes | Yes | Yes | Yes | Yes | No |
| Whitman 1982 | Yes | Yes | Yes | Yes | Yes | Yes | Yes | No |
| Yeager 1984 | Yes | Yes | Yes | Yes | No | No | Yes | No |

The Joanna Briggs Institute Critical Appraisal tools for use in JBI Systematic Reviews, Checklist for Case Reports is based on 8 questions: 1. Were patient’s demographic characteristics clearly described? 2. Was the patient’s history clearly described and presented as a timeline? 3. Was the current clinical condition of the patient on presentation clearly described? 4. Were diagnostic tests or methods and the results clearly described? 5. Was the intervention(s) or treatment procedure(s) clearly described? 6. Was the post-intervention clinical condition clearly described? 7. Were adverse events (harms) or unanticipated events identified and described? 8. Does the case report provide takeaway lessons?

**Supplementary Table 3 – Baseline patient characteristics divided by treatment group**

| Characteristic | Surgery  (n = 55) | No Surgery  (n = 38) | p-value |
| --- | --- | --- | --- |
| Age (years) | 4.5 [10.0-0.47] | 8.00 [11.25-2.56] | **0.036** |
| Female sex | 34/53 (64.2%) | 22/33 (66.7%) | 0.812 |
| Cervical arch laterality |  |  |  |
| *left* | 18/55 (32.7%) | 13/38 (34.2%) | 0.881 |
| *right* | 36/55 (65.5%) | 25/38 (65.8%) | 0.973 |
| *both* | 1/55 (1.8%) | 0/38 (0%) | 0.999 |
| Descending aorta laterality |  |  |  |
| *left* | 51/55 (92.7%) | 30/38 (78.9%) | *0.064* |
| *right* | 4/55 (7.3%) | 8/38 (21.1%) |  |
| Vascular ring | 41/55 (74.5%) | 26/36 (72.2%) | 0.806 |
| Aortic arch associated anomalies | 44/55 (80.0%) | 22/38 (57.9%) | **0.021** |
| *Kommerell’s diverticulum* | 15/55 (27.3%) | 10/38 (26.3%) | 0.919 |
| *Coarctation* | 14/55 (25.5%) | 3/38 (7.9%) | *0.054* |
| *Arch aneurysm* | 10/55 (18.2%) | 1/38 (2.6%) | **0.025** |
| *Hypoplastic* | 6/55 (10.9%) | 0/38 (0%) | *0.078* |
| *Double arch* | 4/55 (7.3%) | 1/38 (2.6%) | 0.645 |
| *Kinking* | 1/55 (1.8%) | 2/38 (2.6%) | 0.565 |
| *Interrupted arch* | 0/55 (0%) | 1/38 (2.6%) | 0.409 |
| Cardiac-associated anomalies | 25/55 (45.5%) | 15/38 (39.5%) | 0.567 |
| *Ventricular septal defect* | 11/55 (20.0%) | 4/38 (10.5%) | 0.264 |
| *Tetralogy of Fallot* | 4/55 (7.3%) | 3/38 (7.9%) | 0.999 |
| *Atrial septal defect* | 2/55 (3.6%) | 3/38 (7.9%) | 0.399 |
| *Bicuspid aortic valve* | 2/55 (3.6%) | 2/38 (5.3%) | 0.999 |
| *Tricuspid atresia* | 2/55 (3.6%) | 0/38 (0%) | 0.512 |
| *Truncus arteriosus* | 1/55 (1.8%) | 2/38 (5.3%) | 0.565 |
| *Double outlet right ventricle* | 0/55 (0%) | 1/38 (2.6%) | 0.409 |
| *Interrupted arch* | 0/55 (0%) | 1/38 (2.6%) | 0.409 |
| Haughton classification |  |  |  |
| *A* | 6/53 (11.3%) | 10/36 (27.8%) | **0.047** |
| *B* | 26/53 (49.1%) | 13/36 (36.1%) | 0.227 |
| *C* | 1/53 (1.9%) | 0/36 (0%) | 0.999 |
| *D* | 15/53 (28.3%) | 10/36 (27.8%) | 0.957 |
| *E* | 3/53 (5.7%) | 2/36 (5.6%) | 0.999 |
| *Not classifiable* | 2/53 (3.8%) | 1/36 (2.8%) | 0.999 |
| Zhong classification |  |  |  |
| *A1* | 13/53 (24.5%) | 9/36 (25.0%) | 0.960 |
| *A2* | 0/53 (0%) | 1/36 (2.8%) | 0.405 |
| *B1* | 5/53 (9.4%) | 2/36 (5.6%) | 0.700 |
| *B2* | 29/53 (54.7%) | 17/36 (47.2%) | 0.487 |
| *B3* | 5/53 (9.4%) | 3/36 (8.3%) | 0.700 |
| *B4* | 1/53 (1.9%) | 3/36 (8.3%) | 0.299 |
| *Not classifiable* | 0/53 (0%) | 1/36 (2.8%) | 0.405 |
| Symptoms |  |  |  |
| *Asymptomatic* | 19/48 (39.6%) | 26/38 (68.4%) | **0.008** |
| *Vascular ring symptoms* | 12/48 (25.0%) | 5/38 (13.2%) | 0.275 |
| *Dyspnea* | 17/48 (35.4%) | 1/38 (2.6%) | **<0.001** |
| *Recurrent RTI* | 7/48 (14.6%) | 3/38 (7.9%) | 0.502 |
| *Dysphagia* | 6/48 (12.5%) | 1/38 (2.6%) | 0.128 |
| *Cough* | 2/48 (4.2%) | 1/38 (2.6%) | 0.999 |
| *Syncope* | 1/48 (2.1%) | 1/38 (2.6%) | 0.999 |
| *Hemiparesis* | 1/48 (2.1%) | 0/38 (0%) | 0.999 |
| *Chest pain* | 1/48 (2.1%) | 0/38 (0%) | 0.999 |
| *Headache* | 0/48 (0%) | 1/38 (2.6%) | 0.442 |
| *Diplopia* | 1/48 (2.1%) | 0/38 (0%) | 0.999 |
| *Palpitations* | 1/48 (2.1%) | 0/38 (0%) | 0.999 |
| Signs |  |  |  |
| *Murmur* | 27/48 (56.3%) | 18/38 (47.4%) | 0.413 |
| *Pulsating mass* | 17/48 (35.4%) | 17/38 (44.7%) | 0.380 |
| *Limbs pressure difference* | 11/48 (22.9%) | 5/38 (13.2%) | 0.279 |
| *Palpable thrill* | 9/48 (18.8%) | 11/38 (28.9%) | 0.266 |
| *Cyanosis* | 7/48 (14.6%) | 5/38 (13.2%) | 0.999 |
| *Stridor* | 4/48 (8.3%) | 1/38 (2.8%) | 0.378 |
| Overall mortality | 4/55 (7.3%) | 3/38 (7.9%) | 0.999 |

**Supplementary Table 4 – Baseline patients characteristics divided by Zhong class**

| Characteristic | Zhong A (n = 25) | Zhong B (n = 67) | p-value |
| --- | --- | --- | --- |
| Age (years) | 9.00 [2.00, 12.00] | 6.00 [0.82, 9.00] | 0.29 |
| Female sex | 13/22 (59.1%) | 42/66 (63.6%) | 0.80 |
| Cervical arch laterality |  |  |  |
| *left* | 21/25 (84.0%) | 9/67 (13.4%) | **<0.001** |
| *right* | 4/25 (16.0%) | 57/67 (85.1%) | **<0.001** |
| *both* | 0/25 (0%) | 1/67 (1.5%) | 0.99 |
| Descending aorta laterality |  |  |  |
| *left* | 22/25 (88.0%) | 58/67 (86.6%) | 0.99 |
| *right* | 3/25 (12.0%) | 9/67 (13.4%) |  |
| Vascular ring | 0/25 (0%) | 67/67 (100%) | **<0.001** |
| Aortic arch associated anomalies | 18/25 (72.0%) | 45/67 (67.2%) | 0.80 |
| *Kommerell’s diverticulum* | 2/25 (8.0%) | 23/67 (34.3%) | **0.02** |
| *Coarctation* | 2/25 (8.0%) | 13/67 (19.4%) | 0.34 |
| *Arch aneurysm* | 9/25 (36.0%) | 2/67 (3.0%) | **<0.001** |
| *Hypoplastic* | 2/25 (8.0%) | 4/67 (6.0%) | 0.66 |
| *Double arch* | 0/25 (0%) | 5/67 (7.5%) | 0.32 |
| *Kinking* | 5/25 (20.0%) | 2/67 (3.0%) | **0.02** |
| *Interrupted arch* | 1/25 (4.0%) | 0/67 (0%) | 0.27 |
| Cardiac-associated anomalies | 8/25 (32.0%) | 28/67 (41.8%) | 0.47 |
| *Ventricular septal defect* | 3/25 (12.0%) | 11/67 (16.4%) | 0.75 |
| *Tetralogy of Fallot* | 2/25 (8.0%) | 5/67 (7.5%) | 0.99 |
| *Atrial septal defect* | 2/25 (8.0%) | 3/67 (4.5%) | 0.61 |
| *Bicuspid aortic valve* | 1/25 (4.0%) | 2/67 (3.0%) | 0.99 |
| *Tricuspid atresia* | 0/25 (0%) | 2/67 (3.0%) | 0.99 |
| *Truncus arteriosus* | 1/25 (4.0%) | 2/67 (3.0%) | 0.99 |
| *Double outlet right ventricle* | 0/25 (0%) | 0/67 (0%) | 0.99 |
| *Interrupted arch* | 1/25 (4.0%) | 0/67 (0%) | 0.27 |
| Symptoms |  |  |  |
| *Asymptomatic* | 14/24 (58.3%) | 31/60 (51.7%) | 0.76 |
| *Dyspnea* | 5/24 (20.8%) | 13/60 (21.7%) | 0.99 |
| *Recurrent RTI* | 2/24 (8.3%) | 7/60 (11.7%) | 0.99 |
| *Dysphagia* | 1/24 (4.2%) | 6/60 (10.0%) | 0.67 |
| *Cough* | 0/24 (0%) | 3/60 (5.0%) | 0.55 |
| *Syncope* | 0/24 (0%) | 2/60 (3.3%) | 0.99 |
| *Hemiparesis* | 1/24 (4.2%) | 0/60 (0%) | 0.29 |
| *Chest pain* | 0/24 (0%) | 0/60 (0%) | 0.99 |
| *Headache* | 0/24 (0%) | 1/60 (1.7%) | 0.99 |
| *Diplopia* | 0/24 (0%) | 1/60 (1.7%) | 0.99 |
| *Palpitations* | 0/24 (0%) | 1/60 (1.7%) | 0.99 |
| Signs |  |  |  |
| *Murmur* | 11/24 (45.8%) | 33/60 (55.0%) | 0.60 |
| *Pulsating mass* | 10/24 (41.7%) | 26/60 (43.3%) | 0.99 |
| *Limbs pressure difference* | 5/24 (20.8%) | 10/60 (16.7%) | 0.75 |
| *Palpable thrill* | 7/24 (29.2%) | 13/60 (21.7%) | 0.66 |
| *Cyanosis* | 1/24 (4.2%) | 9/60 (15.0%) | 0.27 |
| *Stridor* | 0/24 (0%) | 6/60 (10.0%) | 0.18 |
| Underwent surgery | 13/24 (54.2%) | 40/65 (61.5%) | 0.70 |
| *Sternotomy* | 3/13 (23.1%) | 13/65 (20.0%) | 0.72 |
| *Thoracotomy* | 5/13 (38.5%) | 12/65 (18.5%) | 0.14 |
| *Neck* | 0/13 (0%) | 1/65 (1.5%) | 0.99 |
| *Unknown* | 5/13 (38.5%) | 14/65 (21.5%) | 0.27 |
| Underwent surgery for arch | 10/24 (41.7%) | 30/65 (46.2%) | 0.89 |
| *Sternotomy* | 3/10 (30.0%) | 13/30 (43.3%) | 0.71 |
| *Thoracotomy* | 3/10 (30.0%) | 9/30 (30.0%) | 0.99 |
| *Unknown* | 4/10 (40.0%) | 8/30 (26.7%) | 0.45 |
| Overall mortality | 1/25 (4.0%) | 6/67 (9.0%) | 0.67 |
| Mortality after surgery | 0/13 (0%) | 4/40 (10.0%) | 0.56 |
| Mortality after surgery for arch | 0/10 (0%) | 1/30 (3.3%) | 0.99 |

**Supplementary Table 5 - Univariate Firth bias-reduced logistic regression on mortality**

| Variable | Estimate ± SE | p-value | OR (95%CI) |
| --- | --- | --- | --- |
| Age | -0.106927 ± 0.080283 | 0.16 | 0.8985 (0.73944 - 1.0385) |
| Sex | 0.291772 ± 0.748178 | 0.70 | 1.33879 (0.28251 - 5.8737) |
| Cervical arch laterality | 2.106253 ± 1.477313 | *0.06* | 8.2173 (0.94687 - 1079.513) |
| Descending aorta laterality | -1.132294 ± 0.825538 | 0.20 | 0.3222 (0.06769 - 1.9599) |
| Vascular ring | 0.513970 ± 0.936523 | 0.57 | 1.67191 (0.326616 - 16.6385) |
| Aneurysm | -0.787298 ± 1.494328 | 0.56 | 0.4550 (0.00342 - 4.1971) |
| Arch abnormality | -0.517818 ± 0.747853 | 0.49 | 0.5958 (0.13584 - 2.8213) |
| Kommerell diverticulum | 0.251744 ± 0.799630 | 0.76 | 1.2862 (0.21927 - 5.7651) |
| Coarctation | 0.0275879 ± 0.945898 | 0.98 | 1.02797 (0.10217 - 5.3929) |
| Arch hypoplasia | -0.155005 ± 1.516296 | 0.92 | 0.85641 (0.006338 - 8.5738) |
| Double aortic arch | 0.0239532 ± 1.525446 | 0.99 | 1.0242 (0.007531 - 10.6235) |
| Kinking | -0.3101549 ± 1.50956 | 0.83 | 0.7333 (0.00545 - 7.1562) |
| Interrupted arch | 3.721049 ± 1.6827614 | **0.02** | 41.3076 (2.00429 - 6285.375) |
| Concomitant CHD | 0.679982 ± 0.744616 | 0.36 | 1.9738 (0.4534 - 9.3068) |
| Ventricular septal defect | 0.946143 ± 0.817902 | 0.27 | 2.5757 (0.4280 - 12.0422) |
| Atrial septal defect | 0.0239532 ± 1.525446 | 0.99 | 1.0242 (0.007531 - 10.6235) |
| Tetralogy of Fallot | 1.086707 ± 0.9931440 | 0.31 | 2.96449 (0.27904 - 17.7387) |
| Bicuspid aortic valve | 0.2363888 ± 1.538587 | 0.88 | 1.2666 (0.009227 - 13.8406) |
| Tricuspid atresia | 2.6112 ± 1.2241237 | **0.05** | 13.6153 (1.00189 - 187.3441) |
| Truncus arteriosus | 0.499331 ± 1.559040 | 0.76 | 1.6476 (0.01183 - 19.5811) |
| Double outlet right ventricle | 1.369487 ± 1.6766930 | 0.47 | 3.93333 (0.02602 - 80.8674) |
| Symptomatic | 0.959605 ± 0.7943277 | 0.21 | 2.61066 (0.59013 - 15.2095) |
| Vascular ring symptoms | 0.667904 ± 0.8132478 | 0.43 | 1.95014 (0.32612 - 9.0155) |
| Dyspnea | 1.630004 ± 0.7640743 | **0.03** | 5.10389 (1.13372 - 24.9370) |
| Respiratory tract infection | 0.5659577 ± 0.969313 | 0.58 | 1.76113 (0.17033 - 9.8407) |
| Dysphagia | 0.985993 ± 0.9937827 | 0.36 | 2.68047 (0.25209 - 16.0616) |
| Cough | 0.4022855 ± 1.559418 | 0.81 | 1.4952 (0.01073 - 17.7881) |
| Syncope | 0.7514161 ± 1.595591 | 0.66 | 2.1200 (0.01483 - 29.5085) |
| Hemiparesis | 1.274742 ± 1.6770278 | 0.50 | 3.5777 (0.02366 - 73.6064) |
| Headache | 1.274742 ± 1.6770278 | 0.50 | 3.5777 (0.02366 - 73.6064) |
| Chest pain | 1.274742 ± 1.6770278 | 0.50 | 3.5777 (0.02366 - 73.6064) |
| Diplopia | 1.274742 ± 1.6770278 | 0.50 | 3.5777 (0.02366 - 73.6064) |
| Palpitations | 1.274742 ± 1.6770278 | 0.50 | 3.5777 (0.02366 - 73.6064) |
| Murmur | 0.812848 ± 0.7941069 | 0.29 | 2.25432 (0.50971 - 13.1278) |
| Palpable mass | -0.468687 ± 0.794893 | 0.55 | 0.6258 (0.10736 - 2.7737) |
| Pressure difference | -1.25168 ± 1.487577 | 0.32 | 0.2860 (0.002161 - 2.5738) |
| Thrill | -0.301585 ± 0.942228 | 0.74 | 0.7396 (0.07382 - 3.8419) |
| Cyanosis | 1.243837 ± 0.8371824 | 0.16 | 3.4688 (0.56131 - 16.9846) |
| Stridor | -0.412154 ± 1.509996 | 0.77 | 0.6622 (0.00492 - 6.4707) |
| Underwent surgery | -0.120734 ± 0.745334 | 0.87 | 0.88626 (0.20314 - 4.1807) |

CAA = cervical aortic arch; CHD = congenital heart diseases

**Supplementary Table 6 - Univariable Firth’s bias-reduced logistic regression on symptoms**

| Variable | Estimate ± SE | p-value | OR (95%CI) |
| --- | --- | --- | --- |
| Age | -0.072736 ± 0.03904 | *0.06* | 0.9298 (0.8587 - 1.002) |
| Sex | 0.5043896 ± 0.4544427 | 0.27 | 1.6559 (0.6819 - 4.0842) |
| Cervical arch laterality | 0.1178479 ± 0.443136 | 0.79 | 1.1250 (0.4720 - 2.7049) |
| Descending aorta laterality | -0.295435 ± 0.6222202 | 0.63 | 0.7442 (0.2108 - 2.5419) |
| Vascular ring | 0.03318747 ± 0.474109 | 0.94 | 1.0337 (0.4076 - 2.6496) |
| Aneurysm | -0.3170810 ± 0.653248 | 0.63 | 0.7282 (0.1884 - 2.5964) |
| Arch abnormality | 0.4598647 ± 0.4493647 | 0.30 | 1.5838 (0.6609 - 3.8854) |
| Kommerell diverticulum | 0.0168577 ± 0.4655230 | 0.97 | 1.0170 (0.4049 - 2.5425) |
| Coarctation | 1.1191048 ± 0.6747854 | *0.08* | 3.0621 (0.8686 - 13.1985) |
| Arch hypoplasia | 1.2656664 ± 0.9679054 | 0.16 | 3.5454 (0.6214 - 36.4756) |
| Double aortic arch | 2.1090618 ± 1.5272643 | *0.09* | 8.2405 (0.76528 - 1120.902) |
| Kinking | -2.7812536 ± 1.477220 | **0.007** | 0.0619 (0.00047 - 0.53719) |
| Interrupted arch | 1.2123712 ± 1.6468966 | 0.43 | 3.3614 (0.17440 - 496.0775) |
| Concomitant CHD | 0.8022379 ± 0.4353012 | *0.06* | 2.2305 (0.9585 - 5.31754) |
| Ventricular septal defect | 0.7606917 ± 0.5839764 | 0.19 | 2.1397 (0.69735 - 7.16169) |
| Atrial septal defect | -0.8002303 ± 0.999732 | 0.41 | 0.4492 (0.04223 - 2.8677) |
| Tetralogy of Fallot | -0.1781110 ± 0.746178 | 0.81 | 0.8368 (0.1770 - 3.65543) |
| Bicuspid aortic valve | 0.9887095 ± 0.9998377 | 0.30 | 2.6877 (0.42100 - 28.5931) |
| Tricuspid atresia | 1.7475883 ± 1.5640322 | 0.19 | 5.7407 (0.44996 - 800.9005) |
| Truncus arteriosus | 0.6272360 ± 1.055147 | 0.55 | 1.8724 (0.23905 - 21.0866) |
| Double outlet right ventricle | 1.2123712 ± 1.6468966 | 0.43 | 3.3614 (0.17440 - 496.0775) |
| Murmur | -0.0851578 ± 0.422236 | 0.84 | 0.9183 (0.39978 - 2.1049) |
| Palpable mass | -0.0327060 ± 0.428957 | 0.94 | 0.9678 (0.41546 - 2.2478) |
| Pressure difference | 0.2605045 ± 0.5525236 | 0.64 | 1.2975 (0.43665 - 3.9255) |
| Thrill | 0.1160722 ± 0.4986408 | 0.82 | 1.1230 (0.41900 - 3.0131) |
| Cyanosis | 0.6907970 ± 0.6381645 | 0.27 | 1.9953 (0.58481 - 7.5593) |
| Stridor | 0.3734171 ± 0.7462567 | 0.62 | 1.4526 (0.33260 - 6.8666) |
| Underwent surgery | 1.1653919 ± 0.4504622 | **0.008** | 3.2071 (1.34837 - 7.94858) |

CAA = cervical aortic arch; CHD = congenital heart diseases

**Supplementary Table 7 - Univariable Cox regression with Firth’s correction**

| Variable | Estimate ± SE | p-value | OR (95%CI) |
| --- | --- | --- | --- |
| Age | -0.0543167 ± 0.0896815 | 0.50 | 0.9471 (0.76339 - 1.0945) |
| Sex | 0.8282883 ± 0.8187381 | 0.28 | 2.2893 (0.4836 - 10.838) |
| Cervical arch laterality | -1.732285 ± 1.586934 | 0.13 | 0.1768 (0.001355 - 1.4993) |
| Descending aorta laterality | -1.35783 ± 0.8532717 | 0.12 | 0.2572 (0.05689 - 1.47810) |
| Vascular Ring | 0.2048376 ± 1.000529 | 0.82 | 1.2273 (0.24231 - 12.0379) |
| Aneurysm | -1.077935 ± 1.587846 | 0.39 | 0.3402 (0.002607 - 2.8886) |
| Arch abnormality | -0.7511091 ± 0.8559465 | 0.36 | 0.4718 (0.10377 - 2.7246) |
| Kommerell diverticulum | 0.5186067 ± 0.8538429 | 0.52 | 1.6796 (0.2920 - 7.60352) |
| Coarctation | -0.2568362 ± 0.9956159 | 0.78 | 0.7734 (0.07927 - 3.87563) |
| Arch hypoplasia | -0.5305825 ± 1.588874 | 0.70 | 0.5882 (0.00450 - 5.00117) |
| Double aortic arch | -0.430433 ± 1.587186 | 0.76 | 0.6502 (0.004982 - 5.5140) |
| Kinking | 0.1911487 ± 1.593997 | 0.90 | 1.2106 (0.00925 - 10.3745) |
| Interrupted arch | 3.19164 ± 1.193224 | **0.01** | 24.328 (2.22390 - 183.149) |
| Concomitant CHD | 0.8263791 ± 0.8530114 | 0.28 | 2.2850 (0.5057 - 13.1254) |
| Ventricular septal defect | 0.6253368 ± 0.8534166 | 0.45 | 1.8688 (0.32512 - 8.45119) |
| Atrial septal defect | 0.3255366 ± 1.591234 | 0.83 | 1.3847 (0.01059 - 11.816) |
| Tetralogy of Fallot | 1.909918 ± 1.035037 | *0.09* | 6.7525 (0.67543 - 36.6265) |
| Bicuspid aortic valve | 2.139922 ± 1.897403 | 0.28 | 8.4987 (0.06161 - 104.457) |
| Tricuspid atresia | 1.468965 ± 1.001667 | 0.17 | 4.3447 (0.4421 - 22.0567) |
| Truncus arteriosus | 1.472768 ± 1.667747 | 0.41 | 4.3612 (0.03289 - 40.911) |
| Double outlet right ventricle | N/A | N/A | N/A |
| Symptomatic | 0.02146064 ± 0.852665 | 0.98 | 1.0216 (0.22631 - 5.86538) |
| Vascular Ring Symptoms | 0.2648121 ± 0.8547823 | 0.74 | 1.3031 (0.22619 - 5.91113) |
| Dyspnea | 1.200358 ± 0.8524984 | 0.12 | 3.3213 (0.73596 - 19.0617) |
| Respiratory tract infection | 0.1446953 ± 0.9968649 | 0.88 | 1.1556 (0.11829 - 5.80651) |
| Dysphagia | 0.4675008 ± 1.000685 | 0.63 | 1.5960 (0.16259 - 8.08487) |
| Cough | 0.2263899 ± 1.58678 | 0.88 | 1.2540 (0.00961 - 10.6280) |
| Syncope | N/A | N/A | N/A |
| Hemiparesis | 0.6677827 ± 1.597623 | 0.68 | 1.9499 (0.01488 - 16.8031) |
| Headache | N/A | N/A | N/A |
| Chest pain | 1.959887 ± 1.897419 | 0.31 | 7.0985 (0.05146 - 87.2494) |
| Diplopia | 0.9411081 ± 1.627673 | 0.58 | 2.5628 (0.01944 - 22.9295) |
| Palpitations | N/A | N/A | N/A |
| Murmur | 1.301461 ± 0.9950633 | 0.12 | 3.6746 (0.73426 - 35.8289) |
| Palpable mass | -0.7143595 ± 0.9971817 | 0.41 | 0.4895 (0.0500 - 2.46114) |
| Pressure Difference | -1.248836 ± 1.587524 | 0.31 | 0.2868 (0.00219 -2.43360) |
| Thrill | 0.146234 ± 0.9955241 | 0.88 | 1.1574 (0.11865 - 5.79848) |
| Cyanosis | 1.199753 ± 0.8544222 | 0.16 | 3.3192 (0.57656 - 15.0455) |
| Stridor | 0.06651568 ± 1.590372 | 0.96 | 1.0687 (0.008181 - 9.1075) |
| Underwent surgery | -1.627622 ± 0.8555943 | *0.07* | 0.1963 (0.04320 - 1.13236) |

CAA = cervical aortic arch; CHD = congenital heart diseases
